# Who reports high company performance? A quantitative study of Chinese listed companies in the energy industry

**DOI:** 10.1186/s40064-016-3695-y

**Published:** 2016-11-29

**Authors:** Daoyan Guo, Hong Chen, Ruyin Long

**Affiliations:** School of Management, China University of Mining and Technology, No. 1 Daxue Road, Xuzhou, 221116 Jiangsu Province People’s Republic of China

**Keywords:** China, Top managers, Background characteristics, Company performance, Energy industry, Listed companies

## Abstract

In the increasingly competitive environment, top managers’ background characteristics are undoubtedly vital factors for company performance. This study examines whether the performance of Chinese listed companies in the energy industry differs with respect to top managers’ background characteristics and explores the exact distribution interval of top managers’ background characteristics when company performance reaches the highest level. The initial sample was collected from the CSMAR database (2005–2014) for listed companies in the energy industry. After removing the outlier and missing data, the final number of observations was determined as 780. Descriptive statistics were used to investigate the present distribution of top managers’ background characteristics, factor analysis was used to determine the dimensions of company performance, and one-way ANOVA was used to analyze the differences in company performance and its dimensions with respect to top managers’ background characteristics. The findings show that both the age and length of service of top managers present an increasing trend over the years of the study period, whereas the educational level shows no significant changes. The performance of listed companies has three dimensions: profit performance, growth performance, and operating performance. Companies behave differently with regard to their top managers’ background characteristics; when the top manager is 40–45 years old, with a doctoral degree and above, and in the 2nd–3rd year of his service period, his company will achieve a higher level of performance. This study contributes to the growing literature on company performance in the Chinese energy industry by demonstrating the differences in the performance of Chinese listed companies in the energy industry with regard to top managers’ background characteristics, and reaching conclusions on the optimum distribution interval of top managers’ background characteristics when company performance reaches the highest level. This study also provides a valuable reference for organizational reform and performance enhancement, which are urgent problems for the Chinese energy industry.

## Background

Since 2013, falling international energy prices and a weak global economy have posed a severe threat to the development of the energy industry (coal, petroleum, and natural gas) (British Petroleum Group 2014). Chinese listed companies in the energy industry cannot remain immune from this threat (Tang 2015). More seriously, Chinese GDP growth has sharply slowed, and the Chinese government has signaled a strong determination to make the energy structure cleaner (Dang 2015). The worsening international and domestic environments are making the survival of Chinese listed companies in the energy industry a challenge.

In the complicated and competitive markets, human capital is one of the most important intangible assets of listed companies. As representatives of heterogeneous human capital, top managers play a decisive role in the processes of developing companies (Hambrick and Mason 1984; Hamori and Koyuncu 2014; Díaz-Fernández et al. 2015). In Chinese listed companies, the chairman has the top decision-making authority as the delegate of the controlling shareholders, while the chief executive officer (CEO) has the top executive authority with responsibility for the daily business affairs (Michel and Hambrick 1992; Amran et al. 2014; Gao and Hafsi 2015). The top managers, represented by the chairman and CEO, direct the company to achieve strategic goals (Kesner and Sebora 1994; Yoo and Reed 2015). Upper echelons theory shows that, as the result of different background characteristics (e.g., age, gender, educational level, and beliefs), the behaviors of top managers vary significantly, and this influences the decision-making behavior (Allport 1966; Hambrick and Mason 1984; Goll et al. 2008; Pillemer et al. 2014; Ali and Zhang 2015). Company performance is inevitably affected by top managers’ background characteristics (Huang et al. 2010; Amran et al. 2014; Peni 2014).

According to the above introduction, the Chinese energy industry has a strong desire to obtain a better understanding of the effects of top managers’ background characteristics on company performance. Although a considerable number of studies have examined the relationships between the background characteristics of top managers and company performance (e.g., Amran et al. 2014; Peni 2014), few have analyzed the exact distribution interval of the background characteristics of top managers when their company performance reached the highest level. This study focused on Chinese listed companies in the energy industry, which contributes to pushing ahead with the relevant research work. The top managers in this study consisted of the chairman and the CEO, which is in accordance with the definition in most studies (e.g., Amran et al. 2014; Yoo and Reed 2015) and the actual conditions in Chinese listed companies. In addition, as the proportion of female top managers is very small in Chinese listed companies (Gan et al. 2015), and information about top managers’ career experience, beliefs, and other background characteristics is incomplete, this study focused on the age, educational level (EL), and length of service (LoS) for top managers.

Based on the above analysis, this study focuses on the following problems. What is the present distribution of the top managers’ background characteristics in the Chinese energy industry? What are the evaluation dimensions of company performance in this industry? Are there significant differences in company performance and its dimensions with respect to top managers’ background characteristics? Which kinds of top managers’ background characteristics predominate when company performance reaches the higher level?

## Literature review

Since Hambrick and Mason proposed the upper echelons theory in 1984, researchers have conducted much research work on the effect of top managers’ background characteristics on company strategy, organizational performance, etc. Hence, this study mainly reviews the literature concerning the effect of top managers’ background characteristics (comprising age, EL, and LoS) on company performance. Table 1 displays the research status of the relationships between top managers’ background characteristics and company performance.

**Table 1:** Summary of available literature

Content	Conclusion	References	Note
The relationship between age and company performance	Significantly positive correlation	Huang et al. (2010)	–
	Significantly negative correlation	Sun et al. (2006), Tao and Xu (2012) and Moscu (2013)	Main conclusion
	Uncorrelated	Karami et al. (2006)	–
The relationship between EL and company performance	Significantly positive correlation	Shipilov and Danis (2006), Kong and Zhang (2010), Huang et al. (2010) and Tao and Xu (2012)	Main conclusion
	Uncorrelated	Gottesman and Morey (2010) and Yang and Li (2012)	–
The relationship between LoS and company performance	Significantly positive correlation	Bergh (2001)	–
	Significantly negative correlation	Keck (1997) and Huang et al. (2010)	–
	Inverted u-shaped relationship	Hambrick and Fukutomi (1991), Li and Liu (2011) and Luo et al. (2013)	Main conclusion
	Uncorrelated	Tao and Xu (2012)	–

Age is an important variable in terms of top managers’ demographic characteristics (Hambrick and Mason 1984) and it is a double-edged sword. The advantages of young top managers are their vitality, rapid reaction time, and strong learning ability, while the main disadvantage is that they have less management experience. Older top managers have rich management experience, but their weaknesses are their relatively weak cognitive ability (e.g., vigor, logical reasoning ability, and memory) and adaptive capacity (Wiersema and Bantel 1992; Flood et al. 1997; Tihanyi et al. 2000). As shown in Table 1, Huang et al. (2010) suggested that there is a positive relationship between the age of top managers and company performance. Karami et al. (2006) suggested that no relationship exists between age and company performance; however, many studies have empirically verified the negative correlation between the age of top managers and company performance (Sun et al. 2006; Tao and Xu 2012; Moscu 2013; Mesut et al. 2013).

The EL of Top managers is reflected in their cognition level and professional quality, and it determines their abilities to quickly gain and process useful information (Tihanyi et al. 2000). Most researchers believe that a high EL gives top managers the strong cognitive abilities they need to efficiently process information and make precise decisions, which eventually lead to the enhancement of company performance (Hambrick and Mason 1984; Kong and Zhang 2010; Tao and Xu 2012). Thus, there is a positive relationship between top managers’ EL and company performance (Shipilov and Danis 2006; Kong and Zhang 2010; Huang et al. 2010; Tao and Xu 2012).

The LoS can effectively reflect the self-selecting process of a top manager as it exhibits his level of acceptance of the company’s regulations and culture and his loyalty (Pfeffer 1983). The increasing LoS of a top manager enhances organizational cohesion. Similar skills and common cognitive patterns will be easily formed from the theory, beliefs, and experience (Michel and Hambrick 1992; Dikolli et al. 2014), but a long-serving top manager may tend to be satisfied and dependent on the organizational regulations and patterns (Luo et al. 2013). As shown in Table 1, the vast majority of scholars have verified the inverted u-shaped relationship between top managers’ LoS and company performance (Hambrick and Fukutomi 1991; Li and Liu 2011; Luo et al. 2013).

The previous studies have mainly focused on the relationships between top managers’ background characteristics and company performance by using the data of listed companies from all industries, and the results obtained were not completely consistent. Less research has been carried out on Chinese listed companies in the energy industry. Specifically, although Lu et al. (2010), Liu (2010) and a few other scholars have studied the relationships between the average educational level, length of service, age of top management team and company performance, the relevant results are still sketchy and incomplete. Thus, this study has focused on the relationships between top managers’ background characteristics and company performance in the energy industry, and has deeply explored the possible relevant laws.

## Methods

Descriptive statistics were used to investigate the present distribution of top managers’ background characteristics. Factor analysis is a statistical technique that can be used to reduce the number of measured variables and extract common factors from different measured variables. Thus, factor analysis was used to analyze the dimensions of company performance which was measured from the perspective of finance index. The principal component analysis method was used to extract the factors of company performance, and one-way ANOVA was used to analyze the differences in company performance and its dimensions with respect to top managers’ background characteristics.

The initial sample was collected from Chinese listed companies in the energy industry from 2005 to 2014. In accordance with the industry classification standard (2012) established by the China Securities Regulatory Commission, “coal mining and dressing” and “petroleum and natural gas extraction” were selected to constitute the energy industry. After the outlier and missing data were removed, the final number of observations was determined as 780. The age, educational level, length of service of top managers were all derived from the direct disclosure of this information in the company annual reports in the CSMAR database, Furthermore, this information represents the difference between the year of company information disclosure and the year of birth for top managers, the highest educational level when the company performance was reported, and the difference between the year of company information disclosure and the year of tenure initiation. Company performance is a complex concept with multi-dimensional meanings. It encompasses individual performance and organizational performance from the perspective of the appraisal object, and it also covers profit performance, growth performance, operation performance, and development performance. In this study, company performance is defined as the comprehensive performance during a fiscal year measured from vital financial variables (Table 8). These data for these financial variables were derived from the CSMAR database.

## Data analysis

### Descriptive statistics of the sample

**Fig. 1:**
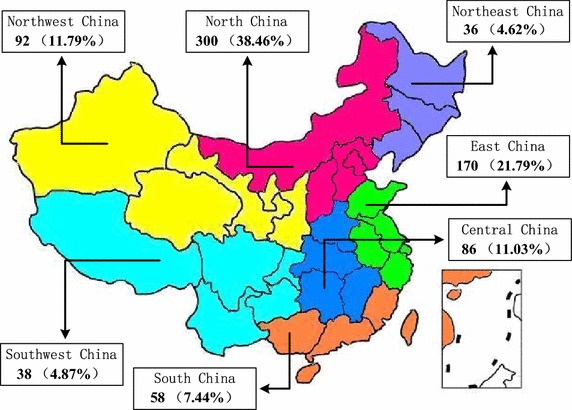
The locations of the companies in the sample

To verify the representativeness of the sample, the location, number of years of operation, number of years since the company was listed, and number of employees were described as follows. Figure 1 is a map showing the location of the sampled companies. It can be seen that the 780 observations were unevenly distributed in China’s seven geographical regions. The number of listed companies in North China and East China account for 60.25% of all companies, which indicates that a large number of the listed companies in the energy industry were distributed in North China and East China. In addition, the descriptions of the companies (e.g., number of years of operation, number of years since the company was listed, number of employees) were shown in Table 2. As shown, the companies have been operating for an average of 13.27 years; they have been listed for an average of 9.33 years; they have an average of 16,192.19 employees, which shows that the size of the companies in the Chinese energy industry is generally large.

**Table 2:** The descriptive statistics of the sample

	N	Mean	Minimum	Median	Maximum	Skewness	Kurtosis
Number of years of operation	780	13.27	3	13	31	0.768	0.555
Number of years listed	780	9.33	1	9	25	0.324	−0.629
Number of employees	780	16,192.19	44	8744	92,738	1.756	2.973

### Analysis of top managers’ background characteristics

Descriptive statistics were used to explore the present distribution of top managers’ background characteristics in the Chinese energy industry.

**Fig. 2:**
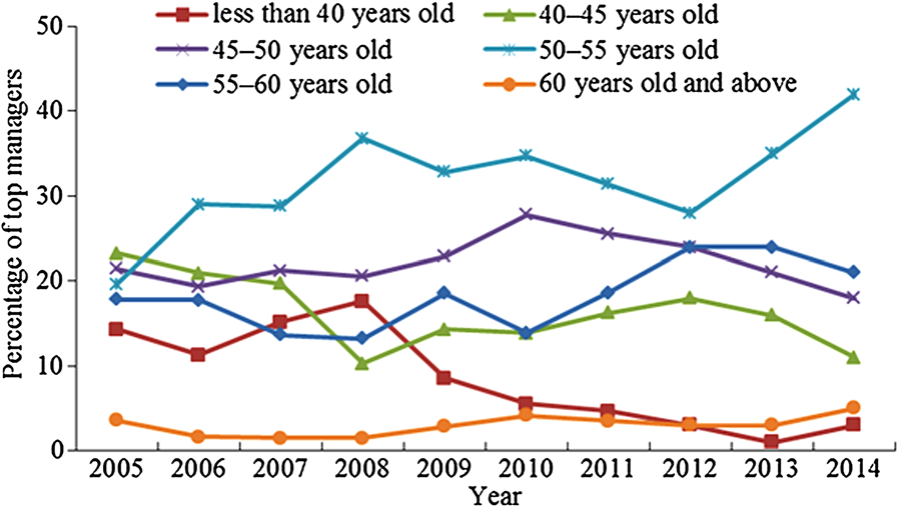
Changing trend of top managers’ age from 2005 to 2014

By referring to the existing research results (Super 1953) and the practical situations of top managers in China, the top managers were divided into six age groups: <40 years old, 40–45, 45–50, 50–55, 55–60, and 60 years old and above. Table 3 describes the present age distribution of these managers for the period 2005–2014. As shown, among the 780 top managers, 58 are <40 years old, 125 are 40–45, 173 are 45–50, 253 are 50–55, 147 are 55–60, and 24 are 60 years old and above. Furthermore, Fig. 2 depicts the changing trend in the age of top managers from 2005 to 2014. Although fluctuations can be observed on the curves for those <40, 40–45, and 45–50 years old, their general trends are downward; in contrast, those 50–55 years old, 55–60 years old, and 60 years old and above show an increasing trend. This shows that the numbers of young top managers are continuously decreasing, whereas the numbers of old top managers are growing. In other words, the average age of the top managers presents a rising trend over the years under review.

**Table 3:** Present distribution of top managers’ age

Year	The number of top managers	Age < 40	40 ≤ Age < 45	45 ≤ Age < 50	50 ≤ Age < 55	55 ≤ Age < 60	60 ≤ Age
2005	56	8	13	12	11	10	2
2006	62	7	13	12	18	11	1
2007	66	10	13	14	19	9	1
2008	68	12	7	14	25	9	1
2009	70	6	10	16	23	13	2
2010	72	4	10	20	25	10	3
2011	86	4	14	22	27	16	3
2012	100	3	18	24	28	24	3
2013	100	1	16	21	35	24	3
2014	100	3	11	18	42	21	5
Total	780	58	125	173	253	147	24

**Fig. 3:**
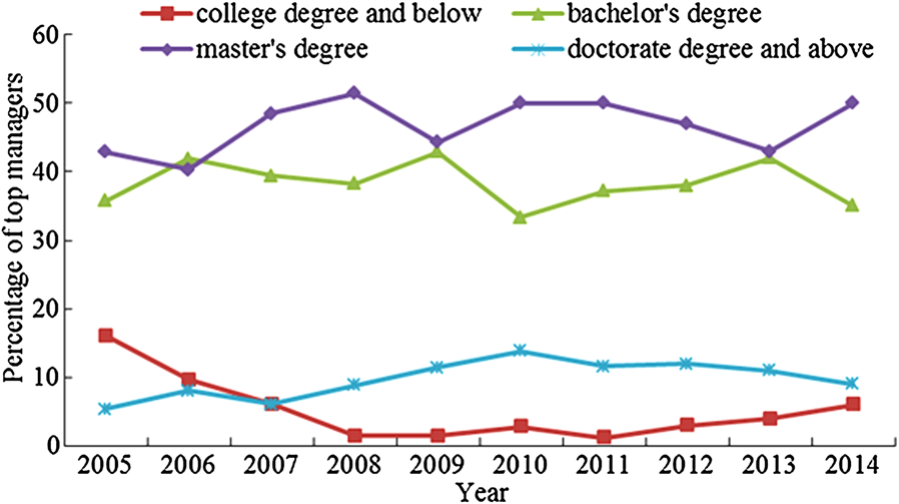
Changing trend in the EL for top managers from 2005 to 2014

China education degree grading standards were used to divide the top managers into four groups: college degree and below, bachelor’s degree, master’s degree, and doctorate degree and above. Table 4 shows the present distribution of the EL of the top managers for 2005–2014. As shown, among the 780 top managers, 37 have a college degree and below, 299 have a bachelor’s degree, 366 have a master’s degree, and 78 have a doctoral degree and above. Furthermore, Fig. 3 depicts the changing trend of top managers’ EL for 2005–2014. It can be seen that those with bachelor’s and master’s degrees account for the largest proportion, while the proportion with doctoral degrees and above is about 10% and the proportion with a college degree and below is lower still. These results illustrate that the top managers generally have bachelor’s and master’s degrees; not as many of them have lower or higher educational levels. There is no significant change in the top managers’ educational levels over the years under review.

**Table 4:** Distribution of top managers’ EL

Year	The number of top managers	College degree and below	Bachelor’s degree	Master’s degree	Doctorate degree and above
2005	56	9	20	24	3
2006	62	6	26	25	5
2007	66	4	26	32	4
2008	68	1	26	35	6
2009	70	1	30	31	8
2010	72	2	24	36	10
2011	86	1	32	43	10
2012	100	3	38	47	12
2013	100	4	42	43	11
2014	100	6	35	50	9
Total	780	37	299	366	78

**Fig. 4:**
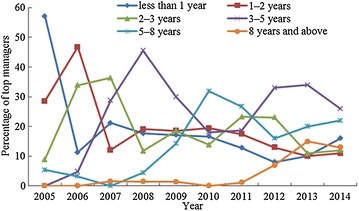
Changing trend of top managers’ LoS from 2005 to 2014

Article 46 of the Company Law of the People’s Republic of China states that “the term of the directors shall be prescribed by the articles of association, provided that each term may not exceed three years. A director may continue to serve in his post if he is re-elected upon the expiration of his term.” The top managers were divided into six groups in respect of their LoS as directors at their company: <1 year, 1–2 years, 2–3 years, 3–5 years, 5–8 years and 8 years and above. Table 5 shows the distribution of the top managers’ LoS in Chinese listed companies in the energy industry for the period 2005–2014. As shown, among the 780 top managers, 134 had a service period of <1 year, for 142 managers it was 1–2 years, for 147 managers it was 2–3 years, for 196 managers it was 3–5 years, for 122 managers it was 5–8 years, and for 39 managers it was 8 years and above. Furthermore, Fig. 4 depicts the changing trend of the LoS of the top managers from 2005 to 2014. Although fluctuations were observed on the curves for the periods of <1 year, 1–2 years, and 2–3 years, their general trends are upwards; in contrast, the groups with lengths of service of 3–5 years, 5–8 years and 8 years and above show a decreasing trend. These results demonstrate that the number of top managers with a short service period is continuously decreasing, whereas the reverse is the case for top managers with a long service period; in other words, the average LoS of the top managers examined presents a rising trend over the period 2005–2014.

**Table 5:** Distribution of top managers’ LoS

Year	The number of top managers	LoS <1 year	1 ≤ LoS < 2 years	2 ≤ LoS < 3 years	3 ≤ LoS < 5 years	5 ≤ LoS < 8 years	8 years ≤ LoS
2005	56	32	16	5	0	3	0
2006	62	7	29	21	3	2	0
2007	66	14	8	24	19	0	1
2008	68	12	13	8	31	3	1
2009	70	12	13	13	21	10	1
2010	72	12	14	10	13	23	0
2011	86	11	15	20	16	23	1
2012	100	8	13	23	33	16	7
2013	100	10	10	11	34	20	15
2014	100	16	11	12	26	22	13
Total	780	134	142	147	196	122	39

### Analysis of company performance

Factor analysis was used to analyze company performance. The principal component analysis was used to extract the components from the perspective of finance. According to the extraction conditions (eigenvalue greater than 1.0 rule), the following four variables needed to be rejected because their factor loadings were <0.5: the current liabilities rate, the asset-liability ratio, Tobin’s q, and the profit growth rate. The factor analysis results of the remaining 16 variables are shown in Table 6. It can be seen that the observed value of Bartlett’s sphericity test was 18,357.654, and the corresponding probability P value was close to 0 (less than the significance level 0.05). This study extracted the constituents of the list that explained most of the variance. Meanwhile, the Kaiser–Meyer–Olkin (KMO) measure was 0.656, which is in the acceptable range (>0.5) (Ranaweera 2016); thus, the variables are suitable for factor analysis.

**Table 6:** KMO and Bartlett’s test

Kaiser–Meyer–Olkin measure of sampling adequacy	0.656
Bartlett’s test of sphericity	
Approx. Chi square	18,357.654
*df*	120
Sig.	0.000

Table 7 explains the total variance. The eigenvalue of the first component was 4.362, the proportion of the variance explained was 27.262%, and the accumulated variance contribution rate of the six extracted factors was 88.163%. Overall, the original variable information loss is less so the factor analysis results are reasonable.

**Table 7:** Total variance explained

Component	Initial eigenvalues			Extraction sums of squared loadings		
	Total	% of variance	Cumulative %	Total	% of variance	Cumulative %
1	4.362	27.262	27.262	4.362	27.262	27.262
2	2.790	17.441	44.703	2.790	17.441	44.703
3	2.085	13.031	57.733	2.085	13.031	57.733
4	1.917	11.983	69.716	1.917	11.983	69.716
5	1.710	10.685	80.401	1.710	10.685	80.401
6	1.242	7.761	88.163	1.242	7.761	88.163
7	0.961	6.007	94.170			
8	0.287	1.796	95.966			
9	0.284	1.776	97.742			
10	0.162	1.013	98.755			
11	0.073	0.455	99.211			
12	0.063	0.396	99.607			
13	0.034	0.210	99.817			
14	0.023	0.142	99.959			
15	0.004	0.025	99.984			
16	0.003	0.016	100.000			

The factors were extracted by principal component analysis

Table 8 shows the component matrix for company performance. It is noteworthy that in exploratory factor analysis, to insure the high efficiency of factor recognition, each extracted latent factor should contain at least three measured variables (Comrey and Lee 1992; Fabrigar et al. 1999; Sun and Zhou 2005). Similarly, some scholars have suggested that the number of measured variables should be 3–5 times the number of extracted latent factors (Hu and Mo 2002; Sun and Zhou 2005). Therefore, this study labeled the extracted four latent factors (3, 4, 5, 6) as one factor so that the factor analysis process of company performance becomes efficient. With respect to their contents: factor 1 mainly explains the five variables (return on equity, earnings before interest and tax ratio, return on assets, cost and expense ratio, main business gross profit ratio) and indicates the number and level of company earnings, so factor 1 was named the company profit performance (CPP). Factor 2 mainly explains the three variables (total assets growth rate, main business growth rate, capital preservation growth rate) and indicates the expansion of the company size and the increase in profit and owner’s equity, so factor 2 was named the company growth performance (CGP). Factors 3, 4, 5, and 6 mainly explain the eight variables (quick ratio, current ratio, equity ratio, debt to tangible assets ratio, cash to net profit ratio, cash to profit ratio, total assets turnover, inventory turnover) and indicate the efficiency and value of the company operating assets, so factors 3, 4, 5, and 6 were named the company operating performance (COP). In conclusion, the company performance has three dimensions: profit performance, growth performance, and operating performance.

**Table 8:** Factor loading matrix for company performance

Factor	Variables	Component					
		1	2	3	4	5	6
Factor 1	Return on equity	*0.891*	−0.190	−0.275	−0.129	−0.048	0.129
	Earnings before interest and tax ratio	*0.876*	−0.037	−0.046	0.269	0.024	0.324
	Return on assets	*0.859*	−0.174	0.001	0.248	0.033	0.351
	Cost and expense ratio	*0.743*	−0.297	0.279	0.334	0.089	−0.043
	Main business gross profit ratio	*0.677*	−0.319	0.297	0.321	0.069	−0.348
Factor 2	Total assets growth ratio	0.392	*0.885*	0.124	−0.086	0.027	−0.112
	Main business growth ratio	0.391	*0.883*	0.117	−0.099	0.038	−0.080
	Capital preservation growth ratio	0.469	*0.840*	0.115	−0.105	0.021	−0.071
Factor 3	Quick ratio	0.070	−0.215	*0.790*	−0.523	0.176	0.118
	Current ratio	0.051	−0.211	*0.774*	−0.545	0.176	0.152
Factor 4	Equity ratio	−0.497	0.241	0.424	−*0.653*	0.123	0.253
	Debt to tangible assets ratio	−0.503	0.253	0.419	−*0.653*	0.120	0.235
Factor 5	Cash to net profit ratio	−0.056	0.015	−0.251	−0.041	*0.880*	−0.076
	Cash to profit ratio	−0.009	0.004	−0.254	−0.026	*0.876*	−0.046
Factor 6	Total assets turnover	−0.120	0.218	0.282	−0.313	−0.083	*0.739*
	Inventory turnover	0.031	0.053	0.113	0.010	−0.224	*0.674*

Italicized values indicate the factor loading value is >0.5, reflecting the items of each of the factors

Calculation formulas (1), (2), (3), and (4) represent the company performance, company profit performance, company growth performance, and company operating performance, respectively. The expressions for the four latent factors should be a linear combination of observed variables (e.g., Macciotta et al. 2006; Fukuda 2011), and they cannot be obtained directly from the output window. The corresponding coefficient of each variable is equal to the factor loading divided by the square root of the corresponding eigenvalues. On this basis, the weight is equal to the corresponding eigenvalue divided by the sum of the extraction factor eigenvalues. The normalized processing is then carried out and the coefficient of each variable in the factor comprehensive model can finally be calculated.

1 $$\begin{aligned} CP & = 0.057X_{1} + 0.135X_{2} + 0.125X_{3} + 0.109X_{4} + 0.080X_{5} + 0.124X_{6} + 0.125X_{7} \\ & \quad + 0.129X_{8} + 0.031X_{9} + 0.028X_{10} - 0.027X_{11} - 0.028X_{12} + 0.030X_{13} + 0.037X_{14} \\ & \quad - 0.001X_{15} + 0.0431X_{16} \\ \end{aligned}$$

2 $$\begin{aligned} CPP & = 0.209X_{1} + 0.205X_{2} + 0.201X_{3} + 0.174X_{4} + 0.159X_{5} + 0.092X_{6} + 0.092X_{7} \\ & \quad + 0.110X_{8} + 0.016X_{9} + 0.012X_{10} - 0.117X_{11} - 0.118X_{12} - 0.013X_{13} - 0.002X_{14} \\ & \quad - 0.028X_{15} + 0.007X_{16} \\ \end{aligned}$$

3 $$\begin{aligned} CGP & = - 0.097X_{1} - 0.019X_{2} - 0.089X_{3} - 0.152X_{4} - 0.164X_{5} + 0.454X_{6} + 0.453X_{7} \\ & \quad + 0.431X_{8} - 0.110X_{9} - 0.108X_{10} + 0.124X_{11} + 0.130X_{12} + 0.008X_{13} + 0.002X_{14} \\ & \quad + 0.112X_{15} + 0.027X_{16} \\ \end{aligned}$$

4 $$\begin{aligned} COP & = - 0.072X_{1} + 0.103X_{2} + 0.116X_{3} + 0.134X_{4} + 0.075X_{5} - 0.003X_{6} + 0.001X_{7} \\ & \quad - 0.002X_{8} + 0.143X_{9} + 0.141X_{10} + 0.049X_{11} + 0.044X_{12} + 0.076X_{13} + 0.083X_{14} \\ & \quad - 0.001X_{15} + 0.114X_{16} \\ \end{aligned}$$

### Descriptive statistics of company performance

Table 9 exhibits the descriptive statistics results of company performance and its dimensions in the Chinese energy industry. As shown in Table 9, the overall mean value for company performance was 0.949, the minimum value was −0.556, the maximum value was 3.715, and the standard deviation was 0.652, which indicate that company performance varies dramatically. Furthermore, among the three dimensions of company performance, the standard deviation (0.306) for the profit performance was more than three times its mean value (0.086), and the standard deviation for growth performance and operating performance were also more than 50% of the mean value, respectively. The statistical results prove that the company performance and its dimensions vary dramatically among the companies examined. The performance levels of some listed companies are high, whereas the others are facing huge survival crises with low levels of company performance.

**Table 9:** Descriptive statistics of company performance

Variable	N	Mean	Minimum	Median	Maximum	SD	SE
CP	780	0.949	−0.556	0.799	3.715	0.652	0.023
CPP	780	0.086	−1.196	0.109	0.911	0.306	0.110
CGP	780	0.959	−0.512	0.923	3.686	0.595	0.021
COP	780	2.312	−0.699	1.766	7.607	1.540	0.055

### Variance analysis of company performance

The differences in company performance and its dimensions were analyzed with respect to top managers’ background characteristics by using a one-way ANOVA (analysis of variance). The results of the homogeneity of variance test showed that the P value was less than alpha (alpha is set at 0.05), which indicated an equal variance is not assumed, so an F test cannot be used. According to the studies by Welch (1951) and Dong (1994), the Welch test was employed to test the significance when using one-way ANOVA to analyze the differences in company performance and its dimensions with respect to the background characteristics of top managers.

Table 10 displays the test results of the differences in company performance and its dimensions with respect to the age of the top managers. As shown, the P value of the Welch test were <0.05, which implies that company performance and its dimensions differ significantly. Further analysis showed that when the top manager is 40–45 years old, company performance (1.144), profit performance (0.188), growth performance (1.195), and operating performance (2.691) were significantly higher than the corresponding performances of other ages.

**Table 10:** Variance analysis of company performance with respect to age

Variable	Age < 40		40 ≤ Age < 45		45 ≤ Age < 50		50 ≤ Age < 55		55 ≤ Age < 60		60 ≤ Age		Welch	Sig.
	Mean	SD	Mean	SD	Mean	SD	Mean	SD	Mean	SD	Mean	SD		
CP	0.710	0.42	1.144	0.73	0.937	0.60	0.929	0.61	0.952	0.71	0.795	0.84	5.436	*0.000*
CPP	0.056	0.20	0.188	0.30	0.086	0.31	0.078	0.34	0.026	0.26	0.101	0.33	4.569	*0.001*
CGP	0.972	0.55	1.195	0.81	0.910	0.58	0.942	0.57	0.912	0.37	0.515	0.52	5.793	*0.000*
COP	1.728	0.99	2.691	1.70	2.265	1.45	2.271	1.42	2.380	1.73	2.096	1.98	5.036	*0.000*

Italicized values indicate the variables have passed the significance test

Table 11 exhibits the test results of differences in company performance and its dimensions with respect to the top managers’ EL. As shown, the P values of the Welch test were <0.05, which implies that company performance and its dimensions differ significantly. Further analysis showed when the top manager has a doctoral degree and above, company performance (1.421), profit performance (0.218), growth performance (1.230), and operating performance (3.428) were significantly higher than the corresponding performances of other educational levels.

**Table 11:** Variance analysis of company performance with respect to EL

Variable	College degree and below		Bachelor’s degree		Master’s degree		Doctorate degree and above		Welch	Sig.
	Mean	SD	Mean	SD	Mean	SD	Mean	SD		
CP	0.680	0.72	0.912	0.62	0.906	0.65	1.421	0.55	17.641	*0.000*
CPP	0.108	0.27	0.096	0.30	0.048	0.32	0.218	0.27	7.558	*0.000*
CGP	0.814	0.84	0.931	0.49	0.938	0.64	1.230	0.54	6.624	*0.000*
COP	1.811	1.62	2.207	1.49	2.210	1.52	3.428	1.34	16.954	*0.000*

Italicized values indicate the variables have passed the significance test

Table 12 exhibits the test results of differences in company performance and its dimensions with respect to the top managers’ LoS. As shown, the P values of the Welch test in company performance, profit performance, and operating performance were <0.05, which implies that the company performance, profit performance, and operating performance differ significantly. The P value (0.206) of the Welch test in company growth performance was larger than 0.05, which implies that there was no significant difference in growth performance. Further analysis showed that when the top manager was at the 2nd–3rd year of his service time, the company performance (1.152), profit performance (0.147), and operating performance (2.751) were significantly higher than the corresponding performance levels for other lengths of service.

**Table 12:** Variance analysis of company performance with respect to LoS

Variable	LoS < 1 year		1 ≤ LoS < 2 years		2 ≤ LoS < 3 years		3 ≤ LoS < 5 years		5 ≤ LoS < 8 years		8 years ≤ LoS		Welch	Sig.
	Mean	SD	Mean	SD	Mean	SD	Mean	SD	Mean	SD	Mean	SD		
CP	0.995	0.62	0.918	0.64	1.152	0.67	0.839	0.59	0.889	0.73	0.877	0.62	4.475	*0.001*
CPP	0.092	0.29	0.053	0.36	0.147	0.32	0.098	0.30	0.040	0.26	0.052	0.22	2.254	*0.047*
CGP	0.972	0.48	1.058	0.65	0.888	0.63	0.936	0.62	0.980	0.62	0.870	0.43	1.452	0.206
COP	2.446	1.57	2.241	1.54	2.751	1.63	2.039	1.31	2.192	1.66	1.198	1.49	4.153	*0.001*

Italicized values indicate the variables have passed the significance test

To summarize, although there was no significant difference in growth performance with respect to the top managers’ LoS, as a general statement, when the top manager is aged 40–45, has a doctoral degree and above, and is in the 2nd–3rd year of his service period, his company will achieve a higher performance level among the Chinese listed companies in the energy industry.

### Interaction analysis of company performance

**Fig. 5:**
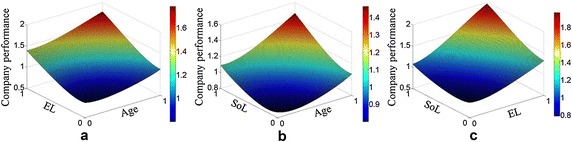
Surface charts of company performance with respect to background characteristics: **a** CP vs. EL and Age; **b** CP vs. SoL and Age; **c** CP vs. SoL and EL

According to the results stated above, the company performance differed significantly with respect to the top managers’ age, EL, and LoS. Accordingly, surface charts of the company performance were drawn to intuitively understand the impact on the company performance of the different background characteristics. Specifically, a top manager who is 40–45 years old was assigned the value 1 and the remaining ages the value 0; a doctoral degree and above was assigned the value 1 and the remaining educational levels the value 0; the 2nd–3rd year of service period was assigned the value 1 and the remaining lengths of service the value 0. Figure 5a depicts the surface chart of the company performance with respect to the age and EL, and the higher point for company performance is 40–45 years old (the value 1) and doctoral degree and above (the value 1). Figure 5b depicts the surface chart of the company performance with respect to age and LoS, and the higher point for company performance is 40–45 years old (the value 1) and the 2nd–3rd year of the service period (the value 1). Figure 5c depicts the surface chart of the company performance with respect to EL and LoS, and the higher point for company performance is a doctoral degree and above (the value 1) and the 2nd–3rd year of the service period (the value 1). In summary, the top managers of high-performing companies tended to be highly localized based on the analysis of the distribution characteristics. The highly performing companies in the Chinese energy industry were mainly in the group of those who were 40–45 years old, with a doctoral degree and above, and in their 2nd–3rd year of service.

### Case analysis

From the perspective of empirical verification, the Pingdingshan Tian’an Coal Mining Corp. Ltd was selected as the case for our analysis.^1^ The research results conformed to the reality of the company. The CEO position at Pingdingshan Tian’an has been consistently held by Xingzi Tu, except for the year 2009, and the company’s annual performance is shown in Table 13. The company was influenced by slower economic growth and decreased energy demand from 2011. The company achieved its highest performance level in 2006; in that year, Xingzi Tu, who has a doctoral degree, was 42 years old and had served with the company for 33 months. This confirmed that when the top manager is 40–45 years old, has a doctoral degree and above, and is in the 2nd–3rd year of his service with the company, his company will achieve a high level of performance among Chinese listed companies in the energy industry.

**Table 13:** Information about Pingdingshan Tian’an Coal Mining Corp

Symbol	Stock number	Year	Background characteristics				CP	CPP	CGP	COP
			Name	Age	EL	LoS (months)				
Pingmei Corp. (Pingmei Tian’an)	601666	2006	Xingzi Tu	42	Doctor	33	*2.393*	*0.399*	*1.788*	*5.923*
		2007	Xingzi Tu	43	Doctor	45	1.930	0.395	1.716	4.512
		2008	Xingzi Tu	44	Doctor	57	2.327	0.578	2.099	5.296
		2009	JianguoYang	52	Master	12	1.881	0.166	1.559	4.820
		2010	Xingzi Tu	46	Doctor	69	1.862	0.294	1.554	4.643
		2011	Xingzi Tu	47	Doctor	81	1.887	0.300	1.521	4.722
		2012	Xingzi Tu	48	Doctor	93	1.975	0.082	1.833	5.078
		2013	Xingzi Tu	49	Doctor	105	1.162	0.138	1.035	2.807
		2014	Xingzi Tu	50	Doctor	117	0.884	0.020	0.950	2.190

## Discussion

From the perspective of evaluating company performance, the performance of listed companies in the Chinese energy industry has three dimensions: CPP, CGP and COP. Obviously, the result is different from that which would apply using mainstream evaluating dimensions such as solvency, turnover ability, profitability, growth ability, and cash-flow ability. This is a very interesting finding. Profit performance measured profit efficiency, which encompassed the amount and percentage of company profit. Growth performance measured the development potential formed by the continuous expansion of the business. Operating performance measured the control efficiency, which indicates the ability to use capital for repaying debt, the turnover, and the ability to convert capital into cash. Hence, evaluating company performance with the dimensions of profit, growth, and operations not only highlights the uniqueness of the energy industry but also provides practical guidance.

From the perspective of top managers’ background characteristics, there are certain relationships between the background characteristics and company performance. Firstly, with respect to age, a considerable number of studies show that the top managers’ age has a significantly negative correlation with the company performance (e.g., Sun et al. 2006). Moreover, Guo (2008) found an inverted U-shaped distribution, but the relationship is between staff age and work performance, therefore, the finding (when the top manager is 40–45 years old, his company will achieve the highest level of performance.) highlights a previously unknown relationship that is important and deserves further study. The finding also indicates that top managers’ optimum age is not as young as might be expected in the Chinese energy industry: accordingly, listed companies should not blindly or mechanically pursue young top managers. Secondly, in respect of the EL, some scholars believe there is no relationship between top managers’ EL and company performance (Gottesman and Morey 2010; Yang and Li 2012). However, a considerable number of studies show that top managers’ EL has a significantly positive correlation with company performance (e.g., Shipilov and Danis 2006; Tao and Xu 2012). In agreement with most studies, this study found that when the top manager has a doctoral degree and above, his company will achieve a higher level of performance. This result indicates that listed companies will achieve higher performance levels when their top manager has a higher EL, whether in the energy industry or other industries, so the result should be highly valued. Thirdly, in respect of the LoS, the previous research mainly verified an inverted U-shaped relationship between top managers’ LoS and company performance (Li and Liu 2011; Xie 2011). Further, Li and Liu (2011) suggested that a 5-year service length is the optimum distribution for company performance, whereas Xie (2011) showed that the optimum LoS is 12 years. This study found that in Chinese energy industry, when the top manager is in his 2nd–3rd year of service with his company, the company will achieve a higher performance level. This finding not only conforms to the results of most previous studies that the company performance exhibits an inverted U-shaped relationship with changing top managers’ LoS (e.g., Li and Liu 2011; Luo et al. 2013), but it also highlights the exact LoS of top managers when company performance reaches the higher level. A possible explanation for the differences between energy industry and others is the science-based nature of the energy industry. In particular, the most significant factors that affect site selection of an oil field, gas field, or coal mine are the resource reserve (e.g., Li and Wang 2008; Rodríguez and Arias 2008). Only an accurate geological exploration can obtain this information. Furthermore, in the energy industry, production is closely related to benefits. Thus, to increase production, mining industry should improve the performance of equipment, and oil and gas industry need to develop enhanced recovery techniques based mainly on geosciences (e.g., Ma et al. 2008; Hartlieb-Wallthor 2014). More importantly, the energy industry must address the problems of human resources. The harsh working conditions in the energy industry require specialized workers, who cannot be trained simply by school education (e.g., Wei et al. 2014). Thus, energy companies need to provide abundant in situ training opportunities so their employees can improve their skills, which would increase labor cost but also enhance the employee value. Therefore, the loyalty of high-value employee is crucial for the energy industry. To secure the loyalty in harsh working conditions, energy industry must keep improving the management mechanism. Thus, innovative ability, the ability to adapt to the environment, the capacity for continuous learning, and cognitive ability are necessary for top managers in energy industry. In conclusion, the science-based nature of the energy industry might present formidable challenges for top managers, thereby resulting in differences between energy industry and others.

## Conclusions

Based on this study, the following conclusions can be stated.

(1) Both the age and LoS of the top managers in the Chinese energy industry present an increasing trend as the years go by, whereas the EL shows no significant changes over that time. (2) The performance of the listed companies in the Chinese energy industry has three dimensions: profit performance, growth performance, and operating performance. (3) The performance achieved by the Chinese listed companies in the energy industry, and their dimensions, vary dramatically; some perform at a high level whereas the others are facing huge survival crises with low levels of company performance. (4) Company performance and its dimensions differ significantly with respect to the top managers’ background characteristics. When the top manager is 40–45 years old, has a doctoral degree and above, and is in the 2nd–3rd year of his service with the company, his company will achieve a high performance level.

## Suggestions

According to the research results, there is a big gap between the present distribution and the optimum value of the top managers’ background characteristics in the Chinese energy industry. In order to enhance company performance, the following suggestions with regard to the top managers’ age, EL, and LoS are proposed.

1. In the selection of top managers, the concept of a high-achieving young management team cannot be simply achieved by decreasing the average age; similarly, high performance will not be guaranteed by setting up an old management team in a listed company in the Chinese energy industry. The young top managers are usually characterized by vitality, rapid reaction, and strong learning ability, and this is why young management teams prevail in China. However, a young management team should be the result of a comprehensive consideration of individual competences. The competent young top manager (40–45 years old according to research results) can be acquired by creating an integrated cultivation system that includes talent mining, selection, reserves, training, and appointments. Specifically, according to research results, top manager candidates should be identified before the age of 35. The cultivation program, including training, learning opportunities, and rotations then needs to begin to build the capacity of candidates. Similarly, high company performance is not the inevitable result of an older top manager. Although the older top managers have rich management experience, their cognitive ability and adaptive capacity are relatively weak. Therefore, listed companies are expected to establish a win–win withdrawal mechanism in order to remove the older top managers’ concerns on their interests and to create the necessary development space for young top managers. This process will promote the long-term development of companies.
2. It is advisable for Chinese listed companies in the energy industry to encourage further study and introduce people with doctorates into the top management team. Due to the intensifying market competition, the continuous learning ability of top managers has become particularly important in this industry. A doctoral degree is usually synonymous with a high cognition level, professional quality, and innovation ability, so a top manager with a doctoral degree can quickly gain and process useful information. Therefore, the listed companies in this industry should encourage their top managers to study for a doctoral degree and introduce a professional manager who has a doctoral degree into the cultivation system mentioned above. Moreover, the present top managers should be positive in their training about the relevant laws, finance, human resources, marketing, and internet information necessary to enrich the knowledge of their young doctorate-level managers and improve their comprehensive ability.
3. Chinese listed companies in the energy industry should pay strict attention to the LoS of their top managers. The LoS shows the professional experience of a top manager and his contribution to the company. It reflects the cognitive and embedded level of knowledge of a top manager about the company’s principles and culture and improves the dependency level of top managers in respect of the company’s organizational pattern and routines. However, it also inhibits the level of organizational reform and strategic innovation of companies. In general, listed companies should stipulate a clear duration for the top managers’ LoS in accordance with the Company Law of the People’s Republic of China. Each term cannot exceed three years but re-election frequently occurs. According to the data for Chinese listed companies in the energy industry from 2005 to 2014, the top managers whose actual LoS exceeded 3 years accounted for 45.77% of the total, which is detrimental to the operations of the company. Hence, the strategy for the re-election of top managers should be seriously considered from a long-term perspective. Listed companies are expected to conservatively assess their top managers whose LoS have exceeded 3 years and consider the impact on the company’s operations to identify the potential risks at an early stage.

## Abbreviations

EL: educational level
LoS: length of service
CP: company performance
CPP: company profit performance
CGP: company growth performance
COP: company operating performance

## Footnotes

1 The reason for this choice is as follows. First, the annual performance of the company has been ranked in the top 5% of the 780 observations. Second, compared to the higher-performance companies (e.g. Yang Quan Coal, Lu’an Environmental Energy, Jingyuan Coal and Electricity Power), the poorer mining conditions of Pingdingshan Tian’an have greatly increased its costs (Tian et al. 2006). After taking the mining conditions into consideration, the 2006 annual performance of Pingdingshan Tian’an becomes more representative.

## References

[CR1] Ali A, Zhang W (2015). CEO tenure and earnings management. J Account Econ.

[CR2] Allport GW (1966). Traits revisited. Am Psychol.

[CR3] Amran NA, Yusof M, Ishak R, Aripin N (2014). Do characteristics of CEO and Chairman influence Government-linked companies performance. Proc Soc Behav Sci.

[CR4] Bergh DD (2001). Executive retention and acquisition outcomes: a test of opposing views on the influence of organizational tenure. J Manag.

[CR5] British Petroleum Group (2014) BP statistical review of world energy. www.bp.com. Accessed 16 Sept 2016

[CR6] Comrey AL, Lee HB (1992). A first course in factor analysis.

[CR7] Dang Y (2015). Research on the energy structure in China under the new normal economy. Appl Energy Technol.

[CR8] Díaz-Fernández MC, González-Rodríguez MR, Simonetti B (2015). Top management team’s intellectual capital and firm performance. Eur Manag J.

[CR9] Dikolli SS, Mayew WJ, Nanda D (2014). CEO tenure and the performance-turnover relation. Rev Account Stud.

[CR10] Dong YH (1994). Provement of equivalent of approximate F’ test and Welch–Aspin test when variance heterogeneity of two groups data. J Math Med.

[CR11] Fabrigar LR, Wegener DT, MacCallum RC, Strahan EJ (1999). Evaluating the use of exploratory factor analysis in psychological research. Psychol Methods.

[CR12] Flood PC, Fong C, Smith KG, O’Regan P, Moore S, Morley M (1997). Top management teams and pioneering: a resource-based view. Int J Hum Resour Manag.

[CR13] Fukuda K (2011). Age–period–cohort decompositions using principal components and partial least squares. J Stat Comput Simul.

[CR14] Gan WY, Xu XX, Lin DJ (2015). Executive gender, power structure and corporate unethical behavior: an empirical test based on PSM data of corporate fraud. Foreign Econ Manag.

[CR15] Gao YQ, Hafsi T (2015). R&D spending among Chinese SMEs: the role of business owners’ characteristics. Manag Decis.

[CR16] Goll I, Johnson NB, Rasheed AA (2008). Top management team demographic characteristics, business strategy, and firm performance in the US airline industry: the role of managerial discretion. Manag Decis.

[CR17] Gottesman AA, Morey MR (2010). CEO educational background and firm financial performance. J Appl Finance.

[CR18] Guo R (2008) The exploring study on the disciplinarian of the performance and staff animal imagery, Dissertation. Beijing Forestry University

[CR19] Hambrick DC, Fukutomi GDS (1991). The seasons of a CEO’s tenure. Acad Manag Rev.

[CR20] Hambrick DC, Mason PA (1984). Upper echelons: the organization as a reflection of its top managers. Acad Manag Rev.

[CR21] Hamori M, Koyuncu B (2014). Experience matters: the impact of prior CEO experience on firm performance. Hum Resour Manag.

[CR22] Hartlieb-Wallthor PV (2014). Chinese coal mining—an analysis with particular regard to the interests of German mining equipment suppliers/Chinesischer Steinkohlenbergbau—Eine Standortbestimmung unter besonderer Berücksichtigung der Interessen deutscher Bergbauzulieferer. Min Rep.

[CR23] Hu ZF, Mo L (2002). The integration of exploratory factor analysis. Psychol Sci.

[CR24] Huang GL, Dong F, Li HQ (2010). The relation of manager characteristics and corporate performance based on perspective of managerial entrenchment. Commer Res.

[CR25] Karami A, Analoui F, Kakabadse NK (2006). The CEOs’ characteristics and their strategy development in the UK SME sector: an empirical study. J Manag Dev.

[CR26] Keck SL (1997). Top management team structure: differential effects by environmental context. Organ Sci.

[CR27] Kesner IF, Sebora TC (1994). Executive succession: past, present & future. J Manag.

[CR28] Kong V, Zhang J (2010). The effect of managerial education and firm-ownership structure: empirical evidence from Chinese listed firms. Chin Econ.

[CR29] Li LH, Liu H (2011). Empirical research on the relationship between CEO tenure and performance. Account Forum.

[CR30] Li H, Wang L (2008). Index system for selection of strategic petroleum reserve bases. Resour Sci.

[CR31] Liu ZS (2010). Restudy on the relationship between educational level and performance of senior management of listed companies in China. Econ Manag J.

[CR32] Lu H, Wang LH, Ke JL (2010). An empirical study on the relationship between top management team content characteristic and performance——based on industry character of China. China Min Mag.

[CR33] Luo X, Kanuri VK, Andrews M (2013). Long CEO tenure can hurt performance. Harv Bus Rev.

[CR34] Ma Y, Zhang S, Guo T, Zhu G, Cai X, Li M (2008). Petroleum geology of the Puguang sour gas field in the Sichuan Basin, SW China. Mar Pet Geol.

[CR35] Macciotta NP, Vicario D, Cappio-Borlino A (2006). Use of multivariate analysis to extract latent variables related to level of production and lactation persistency in dairy cattle. J Dairy Sci.

[CR36] Mesut D, Bilge LE, Veysel A, Serdar Ö (2013). The impact of CEO duality on firm performance: evidence from turkey. Int J Bus Soc Sci.

[CR37] Michel JG, Hambrick DC (1992). Diversification posture and top management team characteristics. Acad Manag J.

[CR38] Moscu RG (2013). The impact of gender and age diversity on company performance. Knowl Horiz Econ.

[CR39] Peni E (2014). CEO and Chairperson characteristics and firm performance. J Manag Gov.

[CR40] Pfeffer J (1983). Organizational demography. Res Organ Behav.

[CR41] Pillemer J, Graham ER, Burke DM (2014). The face says it all: CEOs, gender, and predicting corporate performance. Leadersh Q.

[CR42] Ranaweera HMBP (2016). Perspective of trust towards e-government initiatives in Sri Lanka. SpringerPlus.

[CR43] Rodríguez XA, Arias C (2008). The effects of resource depletion on coal mining productivity. Energy Econ.

[CR44] Shipilov A, Danis W (2006). TMG social capital, strategic choice and firm performance. Eur Manag J.

[CR45] Sun XJ, Zhou ZK (2005). Exploratory factor analysis and its main problems in application. Psychol Sci.

[CR46] Sun HF, Yao ZH, Yan MS (2006). The effect of demographic traits of TMT on performance of textile and IT corporations. Nankai Bus Rev.

[CR47] Super DE (1953). Theory of vocational development. Am Psychol.

[CR48] Tang B (2015). Countermeasures of coal enterprise to market variation. Coal Econ Res.

[CR49] Tao BS, Xu J (2012). The relationship between TMT characteristics and corporate performance: the empirical evidence based on small and medium-sized listed companies. Friendsh Account.

[CR50] Tian SG, Shang GX, Tang X (2006). Chinese coal resources octothorpe shaped distributing pattern: regional differentiation and resources economic geographical division. Coal Geol China.

[CR51] Tihanyi L, Ellstrand AE, Daily CM, Dan RD (2000). Composition of the top management team and firm international diversification. J Manag.

[CR52] Wei X, Li J, Yan Y, Zhang Y (2014). Relationship between occupational burnout and social support for the first line miners. Saf Coal Mines.

[CR53] Welch BL (1951). On the comparison of several mean values: an alternative approach. Biometrika.

[CR54] Wiersema MF, Bantel KA (1992). Top management team demography and corporate strategic change. Acad Manag J.

[CR55] Xie SL (2011) The empirical study of the relationship background characteristics of entrepreneur and organizational performance, Dissertation. University of Electronic Science and Technology of China

[CR56] Yang XY, Li QB (2012). Executive compensation, team characteristics and firm performance sensitivity: the empirical evidence from a-share manufacturing listed companies. Chin Certif Public Account.

[CR57] Yoo JW, Reed R (2015). The effects of top management team external ties and board composition on the strategic choice of late movers. Long Range Plan.

